# Modification and comparison of three *Gracilaria* spp. agarose with methylation for promotion of its gelling properties

**DOI:** 10.1186/s13065-017-0334-9

**Published:** 2017-10-16

**Authors:** Yangyang Gu, Kit-Leong Cheong, Hong Du

**Affiliations:** Department of Biology, Guangdong Provincial Key Laboratory of Marine Biotechnology, STU-UNIVPM Joint Algal Research Center, College of Science, Shantou University, Shantou, 515063 Guangdong PR China

**Keywords:** Agarose, *Gracilaria*, Low-gelling temperature agarose, Physico-chemical properties

## Abstract

In order to improve the gelling properties of agarose, we modified it by methylation. The agarose was prepared from *Gracilaria asiatica*, *G. bailinae*, and *G. lemaneiformis* with alkaline, treated with diatomaceous earth and activated carbon, and anhydrous alcohol precipitation. The methylation reaction process of agarose was performed with dimethyl sulfate while the chemical structure of low-gelling temperature of agarose was also studied by ^13^C-NMR and FT-IR spectra. Results showed that the quality of agarose from *G. asiatica* is optimal. Its electroendosmosis is 0.116, sulfate content is 0.128%, and its gel strength (1.5%, *w/v*) is 1024 g cm^−2^, like those of the Sigma product (A9539). The gelling temperature, melting temperature, and gel strength of the low-gelling temperature agarose is 28.3, 67.0 °C, and 272.5 g cm^−2^, respectively. FT-IR Spectra and ^13^C-NMR spectra also showed that agarose was successfully methylated. Overall, this work suggests that low-gelling temperature agarose may have potential uses as an agar embedding material in various applications such as biomedicine, food, microbiology, and pharmaceutical.

## Introduction

Agar, a mixture of cell-wall polysaccharides including agarose and agaropectin, can be extracted from various species of marine red algae (Rhodophyta) [1]. The predominant agar component, agarose, an electrically neutral polymer, is made up of the repeating unit of agarobiose disaccharide of a 3-*O*-linked β-d-galactopyranose residue, alternating with a 4-*O*-linked 3,6 anhydro-α-l-galactopyranose in linear sequence [2]. The agaropectin is a heterogeneous mixture of smaller molecules that account for lesser amounts of agar. Further, agaropectin is not electrically neutral, due to heavy modifications of sulfate, pyruvate, and methyl side-groups; these chemical substituents are responsible for the varying gel properties of the polysaccharide in aqueous solutions. Due to its non-ionic nature, agarose as aqueous gel has been widely used as culture media and substrates for electrophoresis [3, 4]. Agarose has been used as thickeners in foods, cosmetics, and other conventional uses [5, 6], and can be used for pharmaceutical and cell encapsulation [7, 8].

For all these applications, suitable gelling and melting temperatures of agarose are of particular importance. Biotechnological grade agarose typically has a gelling temperature of about 37 °C and a melting temperature of above 70 °C, which is not favorable for maintaining the activity or integrity of biological reagents. Therefore, we need a low agaropectin content of algae for the preparation of agarose, and via chemical modification to reduce its gelling temperature and obtain the low-gelling form. In general, *Gelidium*-extracted agar typically has better quality, such as higher gel strength, but the high cost plus the gradual exhaustion of natural prairies have prompted a search for alternative sources [9]. We need a kind of algae that can take *Gelidium* for the preparation of agarose. *Gracilaria* (Gracilariales, Rhodophyta), a cosmopolitan genus, has strong adaptability and high speed of growth, which has become one of our options. *G. asiatica*, *G. bailinae*, and *G. lemaneiformis* are rich species of *Gracilaria* algae. In recent years, the *Gracilaria* algae farming industry has developed, e.g., the cultivation area of *G. lemaneiformis* is more than 200,000 acres and production is over 150,000 tons (dried weight) per year in China, providing an excellent substitute for *Gelidium* agar in the industry [10]. However, the quality of agarose from *Gracilaria* species is low, due to high sulfate content. Treatment with sodium hydroxide converts l-galactose-6-sulfate to 3,6-anhydro-l-galactose, and thus greatly improves agarose quality [11, 12]. High quality agarose is obtained by further purification such as isopropanol precipitation, ion-exchange chromatography, and size-exclusion chromatography [13, 14]. Typically, when agarose concentration is 1.0% (*w/v*), high quality agarose has a gel strength of at least 750 g cm^−2^, a gelling temperature of 37 °C, a melting temperature of 85 °C, a sulfate content of 0-0.15% (*w/w*), and an electroendosmosis (EEO) of 0.15 or less [15]. Gel properties include gelling temperature, gel melting temperature, and gel strength with different seaweed sources and extraction conditions [16]. It has also been found that gelling temperature can vary in modified agarose [17].

The aims of this study were to assess which species (*G. asiatica*, *G. bailinae*, and *G. lemaneiformis*) were suitable for agarose preparation; this would involve alkaline treatment with anhydrous alcohol precipitation procedures to obtain good preparation conditions for low-gelling temperature agarose by methylation. Comparison was made of physico-chemical properties of agarose from seaweed to commercially available products of Sigma and Biowest. It might provide more information about FT-IR and ^13^C-NMR spectra related to agarose and low-gelling temperature agarose, and then obtaining the relationship between changes of physico-chemical properties (such as gelling temperature, melting temperature, sulfate content, and EEO) and their structure.

## Experimental

### Materials

Red algae *Gracilaria* (*G. asiatica, G. bailinae*, and *G. lemaneiformis*) were obtained from Chenghai district agar glue factory (Shantou, China). Specimens of *Gracilaria* were harvested in April (2013) in Nan’ao County (23°28′46.23″N and 117°06′24.58″E) in Shantou, China. Three kinds of red algae *Gracilaria* were identified by a corresponding author. For the comparative study, Biowest agarose (Cat. NO. 111860) was purchased from GENE COMPANY LTD. (HK), Commercial agarose (no methylation) (Cat. NO. A9539), low-gelling temperature-agarose (GT: 29.5 ± 1.0 °C, MT: 65.0 ± 0.9 °C, GS: 266.8 ± 5.2 g cm^−2^) (Cat. NO. A9414) while other chemicals were purchased from Sigma-Aldrich Co. LLC. (St. Louis, MO, USA).

### Agarose preparation

Low grade agarose with the higher sulfate content was prepared according to the process specified in the patent [18]. Briefly, red algae *Gracilaria* was boiled in alkaline solution at 90 °C for 2 h, filtered with diatomaceous earth and activated carbon; finally, agarose was dried in air, followed by more drying in the oven at 50 °C for 24 h. Low grade agarose was further purified by using the anhydrous alcohol precipitation. To this end, low grade agarose was dissolved in deionized water (1:50 *w/v*) and autoclaved for 1.5 h at 120 °C. The solution was slowly cooled to about 40 °C with steady stirring. The solution was transferred into a beaker, and anhydrous alcohol (1:4 *v/v*) was added. After thorough mixing and standing for 12 h at room temperature, agarose was obtained by centrifugation at 10,000 rpm min^−1^ at for 30 min at 25 °C, which was dried in the oven at 65 °C for 12 h and ground.

### Agarose methylation

**Fig. 1:**
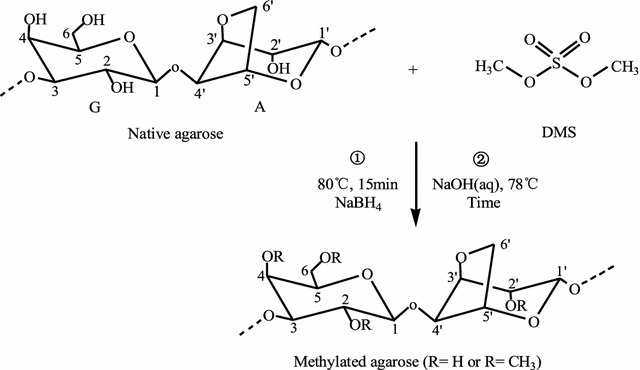
Synthetic routes of methylated agarose

Purified agarose (2 g) was boiled in deionized water (100 mL) for 1 h before adding NaBH_4_ (0.12 g). The reaction mixture was incubated at 80 °C for 15 min with constant stirring. Next, 6.5 mL NaOH (5 mol L^−1^) and 2 mL DMS were added and incubated for 60 min at 78 °C with constant stirring (Fig. 1). After the reaction, the mixture was cooled to 60 °C before being neutralized with 3 mol L^−1^ acetic acid. Methylated agarose was precipitated and dried, and is similar to the preparation of agarose.

### Physical properties

Agarose was powdered and used for measurements of gel strength, gelling temperature, and melting temperature. Also, 1.5% (*w/v*) gel solution was prepared by dissolving agarose in deionized water in an autoclave at 120 °C for 1.5 h. Gel strength was assessed with a Gel Tester (Kiya Seisakusho, Japan). Gelling and melting temperature were measured according to a previous report [19].

### Chemical properties

Sulphate content was determined following the turbidrimetric method, reported by Dodgson and Price (1963) using K_2_SO_4_ as standard. EEO was determined following the modified procedures previously reported [20]. Agarose (0.2 g) was boiled in pH 8.6 TBE buffer (10 mL). The standard test solution consisted of 40 mg mL^−1^ Dextran-700 and 5 mg mL^−1^ bovine serum albumin (BSA). The EEO standards were run at a constant voltage (75 V) for 3 h. EEO (mr) in agarose gel was calculated with the equation: mr = OD/(OD + OA), and OD and OA representing the distance from origin of dextran and albumin.

### DNA electrophoresis

Goldview DNA stain (Takara, China) was loaded into 1% agarose gel in TAE buffer and run at 110 V for 50 min in a standard horizontal electrophoresis unit. DNA was observed under UV illumination, and images were collected immediately after electrophoresis.

### FT-IR spectra

FT-IR spectra of agarose and low-gelling temperature-agarose were recorded with a FT-IR Spectrometer (Nicolet, Rhinelander, WI, USA), in the 4000–400 cm^−1^ range with a resolution of 2 cm^−1^ using KBr pellets.

## ^13^C-NMR

Noise-decoupled ^13^C-NMR spectra of agarose and low-gelling temperature agarose were recorded with a Superconducting Fourier Transform Nuclear Magnetic Resonance Spectrometer (Varian INOVA 500NB, Falls Church, VA, USA) at 125 MHz. The samples were dissolved in D_2_O (50 mg mL^−1^) and analyzed with a 10 mm inverse probe. Spectra were recorded at 70 °C with pulse duration of 15 μs, acquisition time 0.4499 s, relaxation delay 1.55 s, spectral width 29.76 kHz, 3700–3900 scans, using DMSO as the internal standard (ca. 39.5 ppm); the sample was scanned 3700–3900 times.

## Results

### Comparison of agar from *Gracilaria*

The physico-chemical properties of agarose from *G. asiatica*, *G. bailinae*, and *G. lemaneiformis* were measured and compared with those of Bio-west (Logan, UT, USA) and Sigma (St. Louis, MO, USA) (Table 1), showing that gel strength of low-grade agarose was above 750 g cm^−2^, which was close to Biowest agarose. Sulfate content and electroendosmosis of it was higher than Biowest and Sigma, such that alkaline hydrolysis treatment cannot completely remove negative charge groups.

**Table 1:** Physico-chemical properties of agaroses from *G. asiatica*, *G. bailinae*, *G. lemaneiformis*, Sigma, and Biowest

Agarose	GT^a^ (°C)		MT (°C)		GS (g cm^−2^)		SC (%)		EEO	
	C	T	C	T	C	T	C	T	C	T
*G. asiatica*	38 ± 1.2	37 ± 0.3	88 ± 0.8	88 ± 1.5	872 ± 15.8	1024 ± 17.0**	0.17 ± 0.01	0.13 ± 0.02*	0.16 ± 0.005	0.12 ± 0.002*
*G. bailinae*	39 ± 0.8	38 ± 0.3	89 ± 1.0	89 ± 0.5	879 ± 26.9	1003 ± 13.6**	0.20 ± 0.01	0.17 ± 0.02*	0.18 ± 0.004	0.16 ± 0.003
*G. lemaneiformis*	37 ± 0.8	37 ± 0.3	89 ± 1.0	92 ± 0.8	896 ± 23.2	1008 ± 21.6**	0.18 ± 0.02	0.15 ± 0.01*	0.17 ± 0.004	0.15 ± 0.003
Biowest	38 ± 0.8		93 ± 1.9		878 ± 18.1		0.15 ± 0.01		0.13 ± 0.002	
Sigma	37 ± 0.9		92 ± 0.6		1127 ± 23.6		0.12 ± 0.01		0.11 ± 0.003	

Results are expressed as mean ± standard deviation (*n* = 3). Statistically different * *p* < 0.05, ** *p* < 0.01 vs control

*GT* gelling temperature, *MT* melting temperature, *GS* gel strength, *SC* sulfate content, *EEO* electroendosmosis, *C* control group, *T* treatment group

**Fig. 2:**
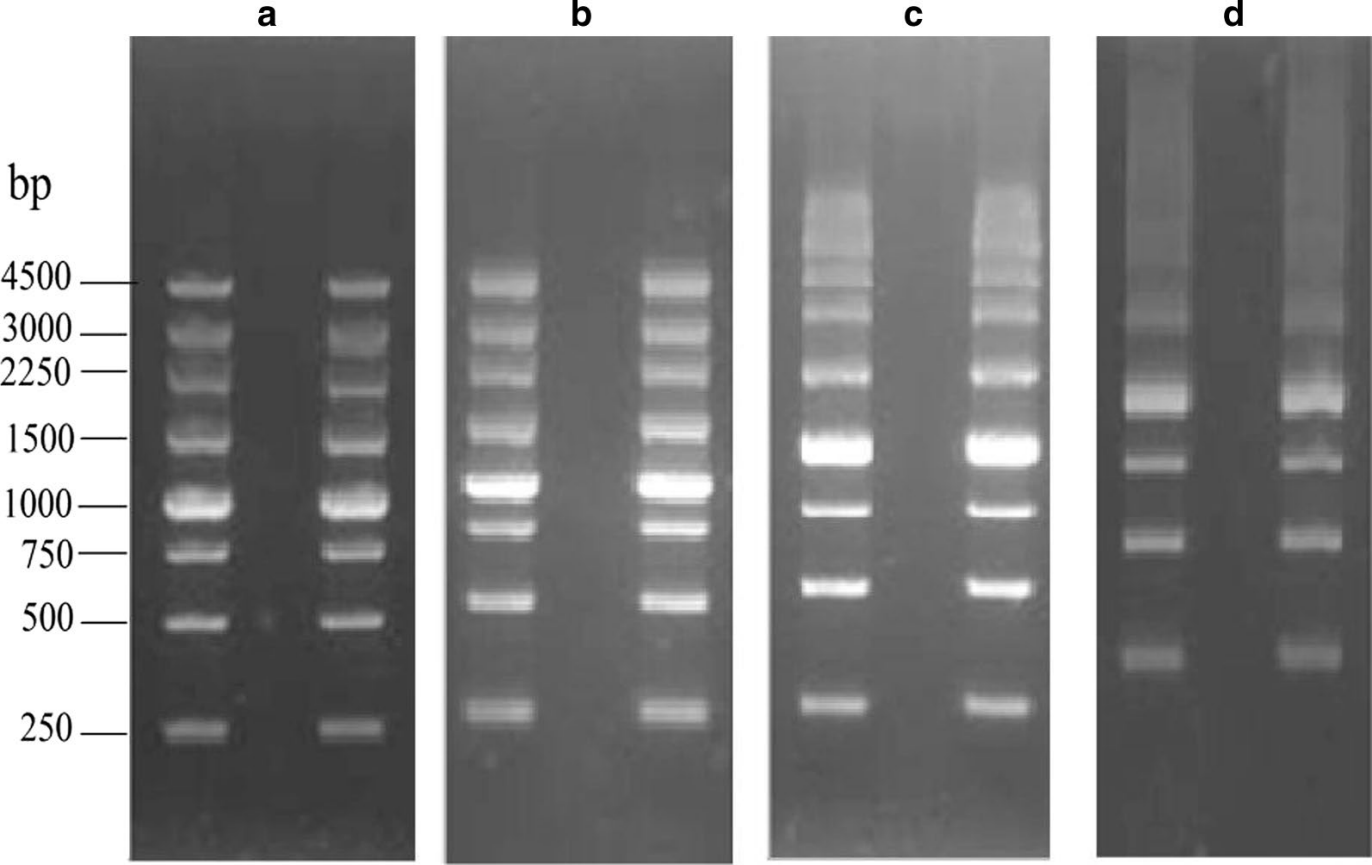
Agarose gel electrophoresis patterns of DNA. Agarose from **a** Biowest, **b** *G. asiatica*, **c** *G. lemaneiformis*, and **d** *G. bailinae.* The gels were exposed to UV light and the picture were taken with a gel documentation system

After treating with anhydrous alcohol, sulfate content and electroendosmosis decreased while gel strength increased in purified agarose (Table 1). Agarose from *G. asiatica* showed the greatest improvement for these parameters after alcohol treatment; however, no significant difference in gelling and melting temperatures (*p* > 0.05) was found. Gel strength of purified agarose from *G. asiatica* (1024 ± 16.8 g cm^−2^) was higher than that of Biowest agarose (878 ± 18.1 g cm^−2^), but it was lower compared Sigma agarose (1127 ± 23.6 g cm^−2^). The sulphate content (0.13 ± 0.02%) and EEO (0.12 ± 0.002) of purified agarose from *G. asiatica* were lower than that of Biowest agarose. The quality of prepared agarose is higher than reported results [21]. Consistently, a DNA electrophoresis experiment showed that eight DNA bands were clearly distinguishable from agarose gel prepared (Fig. 2), indicating that *G. asiatica* agarose gel had higher intensity and better DNA detection sensitivity than agarose from *G. lemaneiformis* and *G. Bailinae*.

### Modification of agarose with methylation

**Fig. 3:**
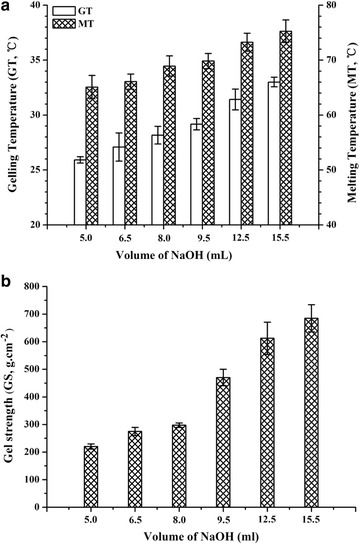
Effect of NaOH aqueous on **a** gelling temperature, melting temperature, and **b** gel strength of agarose. Values are mean ± SD (*n* = 3)

To optimize the methylation condition, NaOH solution in different quantities (5.0–15.5 mL) and 2 mL of DMS were added to the reaction for 75 min. The gelling and melting temperatures and gel strength were positively correlated with the amount of added NaOH (Fig. 3); at 6.5 mL NaOH, the gelling temperature (27 °C) and gel strength (288 g cm^−2^) were 2.5 °C lower and 21.2 g cm^−2^ higher, respectively, than Sigma low-gelling temperature agarose (A9414).

**Fig. 4:**
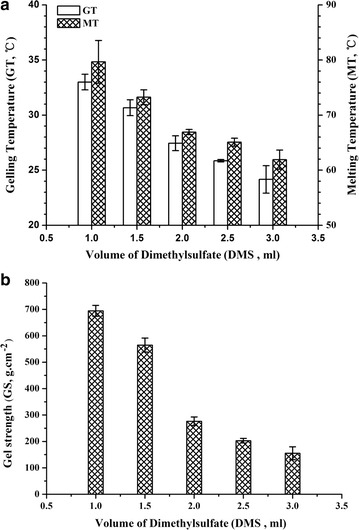
Effect of DMS aqueous on **a** gelling temperature, melting temperature, and **b** gel strength of agarose. Values are mean ± SD (*n* = 3)

DMS in different quantities (1–3 mL) and 6.5 mL of NaOH were added to the reaction for 75 min. The gelling temperatures, melting temperature, and gel strength were negatively correlated to the added DMS (Fig. 4), and at 2.0 mL DMS, the gelling temperature (27 °C), melting temperature (66.9 °C), and gel strength (276 g cm^−2^) were superior to agarose produced at 1 or 3 mL of DMS.

**Fig. 5:**
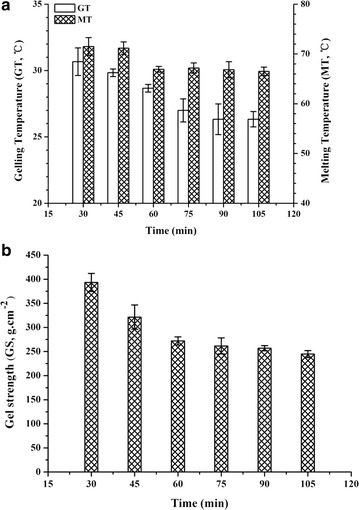
Effect of reaction time on **a** gelling temperature, melting temperature, and **b** gel strength of agarose. Values are mean ± SD (*n* = 3)

We tested the reaction time from 30 to 105 min (Fig. 5). At 60 min, the gelling temperature and melting temperature declined to 28 and 67 °C, respectively. The gel strength was 272 g cm^−2^ and stronger than Sigma low-gelling temperature agarose. The reaction with a recipe of 2 g agarose, 6.5 mL NaOH (5 mol L^−1^), 2 mL DMS, and a reaction time of 60 min produces the most desirable product.

### Chemical properties of methylated agarose

**Fig. 6:**
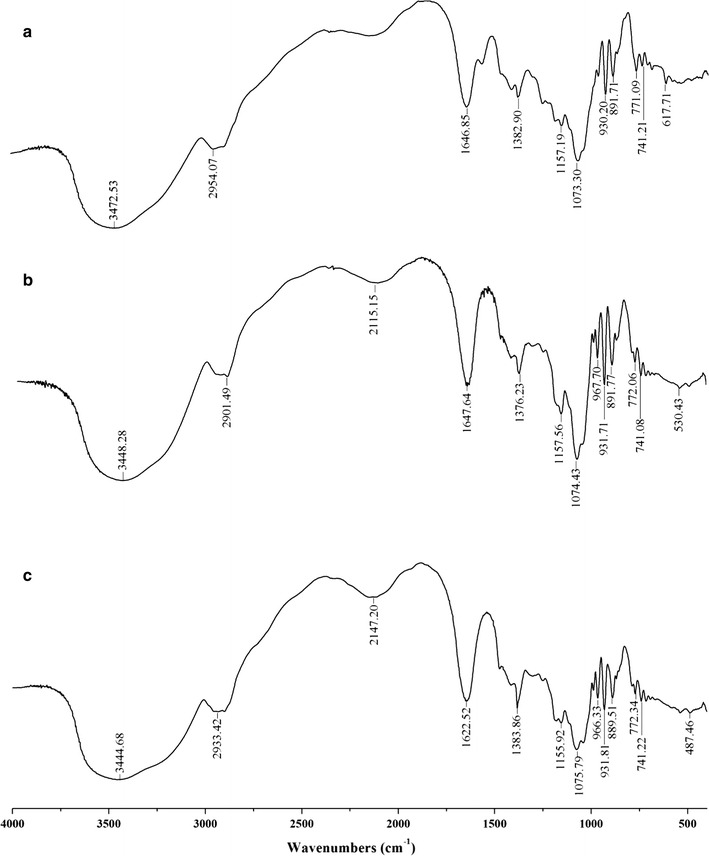
Fourier transforms infrared spectra of **a** *G. asiatica* agarose, **b** *G. asiatica* methylated agarose, and **c** Biowest agarose

FT-IR spectra (Fig. 6) shows no absorption was found in the region of 850–820 cm^−1^, corresponding to C–O–S stretching, and indicating the absence of C4, and C6-sulphate in the galactopyranose moiety. The peak at 820–772 cm^−1^ was sharper than Biowest agarose, demonstrating that agarose from *G. asiatica* had a higher purity. The peak at 930 cm^−1^ was indicative of 3,6-AG residues being sharper and deeper than Biowest agarose, suggesting that agarose from *G. asiatica* had a higher purity, and that negatively charged groups of agar polysaccharides were effectively removed. The huge peak at 3450 cm^−1^ indicated that agarose had a large number of hydroxyl groups. The FT-IR spectra of metylated agarose indicated they have the same carbon skeleton structure with the purified agarose. The spectra experienced a significant change with the peak at 1650 cm^−1^ splitting into two peaks at 1650 and 1566 cm^−1^, and increasing to about 820 cm^−1^ in the methylated agarose. The FT-IR spectra of purified agarose from *G. asiatica* were in agreement with Biowest agarose.

**Fig. 7:**
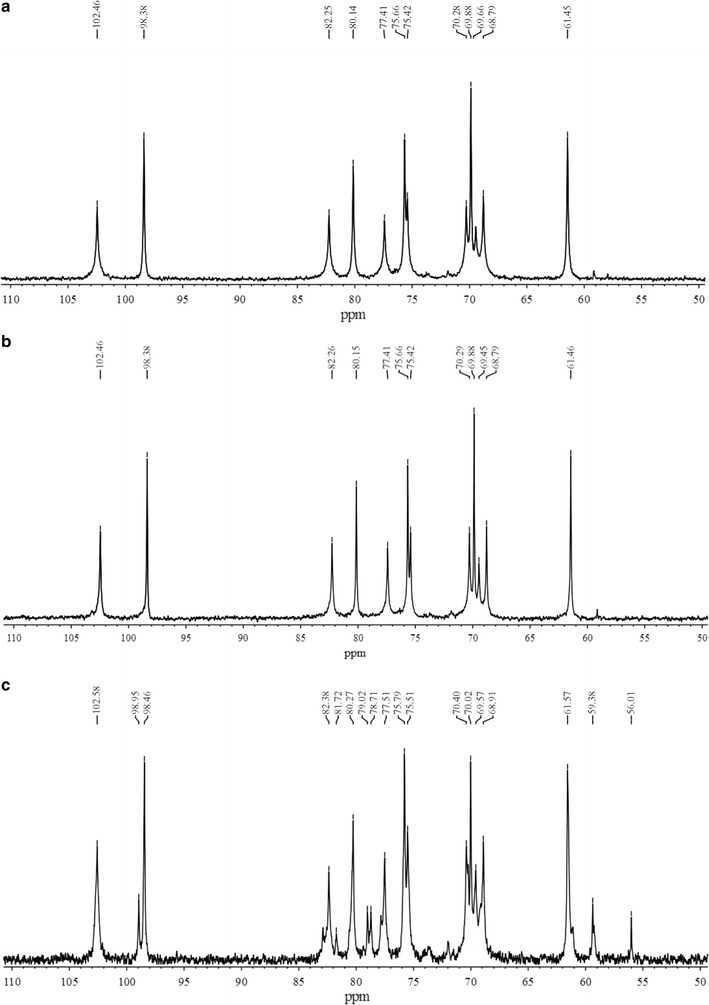
^13^C-NMR spectra of **a** *G. asiatica* agarose, **b** *G. asiatica* methylated agarose, and **c** Biowest agarose

The ^13^C-NMR spectra of agarose samples were presented in Fig. 7 and Table 2. The chemical shifts of the 12 carbon atoms of the disaccharide repeating units of agaroses were comparable with the reported Sigma agarose in the literature [22] (Table 2). The signals at 102.45, 70.28, 82.25, 68.79, 75.42, and 61.45 ppm corresponded to the 3-linked units, while the signals at 98.38, 69.88, 80.14, 77.41, 75.66, and 69.66 ppm corresponded to the 4-linked units. Purified agarose from *G. asiatica* had identical spectra as the agarose from Sigma, while methylated agarose had two additional large -OCH_3_ peaks at 59.2 and 56.01 ppm, with some other new peaks at 98.95, 81.72, 79.02, and 78.71 ppm, showing that NMR spectra from carbon atoms are sensitive to the methylation. Methylation caused the changes of the chemical shift of the adjacent carbon atoms, the effect being from 0.08 to 0.20 ppm (Table 2). All of these results suggested that methylated agarose was successfully synthesized.

**Table 2:** ^13^C-NMR chemical shift of methylated agarose from *G. asiatica* and agarose from *G. asiatica*, Biowest, and Sigma

Agarose	Unit	^13^C chemical shifts (ppm)						
		C1	C2	C3	C4	C5	C6	–OCH_3_
*G. asiatica*	G	102.45	70.28	82.25	68.79	75.42	61.45	–
	A	98.38	69.88	80.14	77.41	75.66	69.66	
*G. asiatica* (methylated)	G	102.58	70.40	82.38	68.91	75.51	61.57	59.38, 56.01
	A	98.46	70.02	80.27	77.51	75.79	69.57	
Biowest	G	102.46	70.29	82.26	68.79	75.42	61.46	–
	A	98.38	69.88	80.15	77.41	75.66	69.45	
Sigma	G	102.23	70.00	81.96	68.49	75.10	61.16	–
	A	97.92	69.75	79.91	77.03	75.40	69.14	

## Discussion

High quality agarose can be obtained with NaOH treatment and anhydrous alcohol precipitation procedures to remove sulfate and pyruvate residues. Agarose prepared from *Gracilaria dura* by alkali treatment has a residual sulfate content of 0.25% [22]. Further treatment with isopropyl alcohol precipitation reduces the sulfate content to 0.14% in agarose prepared from *G. amansi* [1]. In this study, we used the anhydrous alcohol precipitation method, as it is a more environmentally-friendly process; anhydrous alcohol can be recycled during the industrial agarose preparation.

The method of NaOH treatment and anhydrous alcohol precipitation was applied to agarose preparation from *Gracilaria* (*G. asiatica*, *G. bailinae*, and *G. lemaneiformis*). *G. asiatica* had more carbohydrates and less ash than *G. lemaneiformis* (Table 3), which may explain the higher quality of agarose prepared from *G. asiatica*. The molecular weight of agarose, with none of the other substituents, showed a gel strength related to the content of the sulfate residue, reduced the amount of sulfate residue, and increased the purity of agarose and the content of 3,6-anhydrogalactose [16]. The content of 3,6-anhydrogalactose related to the gel strength, the higher content of the 3,6-anhydrogalactose, and the greater the gel strength. However, the gel strength of agarose among the tested species (*G. asiatica*, *G. bailinae*, *G. lemaneiformis*) was not significantly different. The literature had reported that different growth environments, as well as the content of agaropectin being different, included molecular weights of different agarose being different as well [22]. These factors would affect the gel strength, as the lower the molecular weight of agarose, the lower the gel strength. Changes of electroendosmosis were in conformity with the changes of sulfate residue present on the agarose, but it was necessary to clarify electroendosmosis reduction, not only related to the sulfate residue content, but also to the loss of agar of other negatively charged groups.

**Table 3:** Determination and comparison of the proximate composition between *G. asiatica* and *G. lemaneiformis*

Species	Content (%, dry weight)				
	Crude protein	Carbohydrate	Crude fat	Crude fiber	Ash
*G. asiatica*	18.6	61.8	0.4	6.2	13.0
*G. lemaneiformis*	19.1	43.8	0.5	4.8	28.7

Based on the best reaction conditions, the gelling and melting temperature of methylated agarose is lower and higher than Sigma’s product (A9414), respectively. This is due to –OH of Sigma’s product being modified by hydroxyethyl. To our knowledge, the optimization of agarose from *G. asiatica* methylated by using DMS has not been reported. By using less NaOH, DMS, and time during the preparation of methylated agarose, industry operation costs can be reduced. This methylation method of agarose with DMS is safe, simple, convenient, and suitable for industrial application.

In FT-IR spectra of both the prepared agarose from *G. asiatica* and the Biowest agarose, a clear peak at about 3450 cm^−1^ corresponding to –OH stretching was detected. However, the hydroxy peak of methylated agarose at ~ 3450 cm^−1^ did not apparently disappear or decrease, and the –OCH_3_ peak at 2950 cm^−1^ was not an obvious enhancement, indicating –OH of agarose was not completely methylated. Further, –CH_3_ can be directly connected to pyranoses of agarose, leading to the C–O stretch vibration peak split (the peak at 1650 cm^−1^ splits into two peaks at 1650 and 1566 cm^−1^). ^13^C-NMR spectra of prepared agarose only have 12 signals of chemical shift, no chemical shift of carbon atomic agaropectin (101.6, 69.3, 71.2, 79.1, 70.2, and 67.9 ppm) and starch polysaccharide (100.7, 72.7, 74.3, 78.7, 72.5, and 62.2 ppm). These results indicated that the agaropectin and starch polysaccharide in the agar have been removed [23]. In the ^13^C-NMR spectra of methylated agarose, three carbon atoms A1 (98.46 ppm), G3 (82.20 ppm) and A4 (77.51 ppm) appear as distinct small peak signals, possibly due to the presence of –OCH_3_ groups in methylated agarose; this results in anisotropy around the three carbons. FT-IR and ^13^C-NMR spectra correspond to changes of physical properties of methylated agarose.

## Conclusion

In this study, electroendosmosis of preparation agarose from *G. asiatica* was 0.12, sulfate content was 0.13% and gel strength (1.5%, *w/v*) was 1024 g cm^−2^. Low-gelling temperature agarose was prepared successfully. The gelling temperature, melting temperature, and gel strength of the low-gelling temperature was 28.3, 67.0 °C, and 272.5 g cm^−2^, respectively. FT-IR Spectra showed the peak of methylated agarose at around 1650 cm^−1^ split into 1650 and 1566 cm^−1^ with two peaks. ^13^C-NMR spectra of methylated agarose had two clear signals of –OCH_3_ at 59.38 and 56.01 ppm. *G. asiatica* is more appropriate for agarose preparation, as methylated agarose also has good features. This methylated agarose is beneficial for the future application in biomedical, food, microbiology and pharmaceutical areas.
